# Preservation of Language Processing and Auditory Performance in Patients With Disorders of Consciousness: A Multimodal Assessment

**DOI:** 10.3389/fneur.2020.526465

**Published:** 2020-12-21

**Authors:** Stefania Ferraro, Anna Nigri, Ludovico D'Incerti, Cristina Rosazza, Davide Sattin, Davide Rossi Sebastiano, Elisa Visani, Dunja Duran, Giorgio Marotta, Greta Demichelis, Eleonora Catricala', Sonja Kotz, Laura Verga, Matilde Leonardi, Stefano Cappa, Maria Grazia Bruzzone

**Affiliations:** ^1^School of Life Science and Technology, MOE Key Laboratory for Neuroinformation, University of Electronic Science and Technology of China, Chengdu, China; ^2^Neuroradiology Department, Fondazione IRCCS Neurologico Carlo Besta, Milan, Italy; ^3^Neurology, Public Health, Disability Unit and Coma Research Centre, Fondazione IRCCS Neurologico Carlo Besta, Milan, Italy; ^4^Department of Neurophysiology and Diagnostic Epileptology Unit, Fondazione IRCCS Neurologico Carlo Besta, Milan, Italy; ^5^Department of Nuclear Medicine, Fondazione IRCCS Ca' Granda Ospedale Maggiore Policlinico, Milan, Italy; ^6^Department of Psychology, Scuola Universitaria Superiore, Pavia, Italy; ^7^Department of Psychology, Maastricht University, Maastricht, Netherlands; ^8^IRCCS Mondino Foundation, Pavia, Italy

**Keywords:** disorders of consciousness, magnetic resonance imaging, brainstem auditory evoked potentials, positron emission tomography, language processing

## Abstract

The impact of language impairment on the clinical assessment of patients suffering from disorders of consciousness (DOC) is unknown or underestimated and may mask the presence of conscious behavior. In a group of DOC patients (*n* = 11; time post-injury range: 5–252 months), we investigated the main neural functional and structural underpinnings of linguistic processing, and their relationship with the behavioral measures of the auditory function using the Coma Recovery Scale-Revised (CRS-R). We assessed the integrity of the brainstem auditory pathways, of the left superior temporal gyrus and arcuate fasciculus, the neural activity elicited by passive listening of an auditory language task, and the mean hemispheric glucose metabolism. Our results support the hypothesis of a relationship between the level of preservation of the investigated structures/functions and the CRS-R auditory subscale scores. Moreover, our findings indicate that patients in minimally conscious state minus (MCS−): (1) when presenting the *auditory startle* (at the CRS-R auditory subscale) might be aphasic in the receptive domain, being severely impaired in the core language structures/functions; (2) when presenting the *localization to sound* might retain language processing, being almost intact or intact in the core language structures/functions. Despite the small group of investigated patients, our findings provide a grounding of the clinical measures of the CRS-R auditory subscale in the integrity of the underlying auditory structures/functions. Future studies are needed to confirm our results that might have important consequences for the clinical practice.

## 1. Introduction

Severe brain injury might result in disorders of consciousness (DOC), a spectrum of conditions comprising coma, vegetative state (VS), and minimally conscious state (MCS). Coma, characterized by the complete loss of arousal, typically results into either VS or MCS in few weeks after the insult. VS is behaviorally characterized by the re-emersion of spontaneous eye-opening associated with the absence of evidence of awareness of the self or of the environment, while MCS by minimal and fluctuating levels of awareness (1).

The assessment of diagnosis in patients with DOC mainly relies on testing for the presence of conscious behavior through standardized behavioral scales. Among these, the Coma Recovery Scale-Revised (CRS-R) (2) has a well-recognized clinical validity. (3) The presence of cognitive deficits, however, may mask the presence of conscious behaviors also when using these standardized approaches (4) leading to possible misdiagnosis. In this context, aphasia is one of the main confounding factors in the behavioral assessment of these patients (4): among 13 CRS-R items (2) used to classify DOC patients in MCS, almost 80% relies on the presence of preserved language comprehension. A proof of concept that language disorders might lead to misdiagnosis in DOC patients showed that a consistent percentage of fully aware patients with aphasia (up to 54%) did not reach the maximal CRS-R total score and that patients with global aphasia were prone to have a diagnosis underestimating their level of consciousness (5).

The incidence of severe language disorders in DOC patients is unknown. Aphasia, however, is a common outcome in severe traumatic brain injury (up to 50% of cases) and stroke (up to 30% of cases) (6–9). It is therefore likely that language disorders may contribute to the clinical picture of patients with DOC caused by these acute events. The presence of widespread cortical damage in patients with DOC caused by anoxic brain injury (10) allows us to infer that severe language impairment may be a common feature also in this case. The mediation role of linguistic function on CRS-R may also explain neuroimaging findings showing a positive correlation between the anatomical and functional integrity of the left hemisphere (language dominant in 80–95% of individuals) and the clinical status (11, 12).

The need to establish the level of residual language processing in DOC patients has become very relevant in these last years in relation to the proposed subcategorization of MCS patients based on the presence (patients categorized as MCS+), or absence (patients categorized as MCS−), of command following, or intelligible verbalization or yes/no responses (verbal or gestural) as detected at behavioral level (13, 14). Interestingly, the investigations of the neurofunctional underpinnings of this sub-categorization showed a disconnection of the Broca's area (14) within the language network in MCS− patients and that MCS+ patients had higher glucose metabolism in the left fronto-temporal-parietal regions than MCS−. Similarly, a recent study found higher functional connectivity in the language control network in MCS+ than in MCS− patients (15). Overall, there is imaging evidence of more severe involvement of language areas in MCS− patients than in MCS+, suggesting that the level of language processing may play a central role in this clinical sub-categorization (14, 15).

In our previous study, we investigated the neurofunctional markers of language processing in DOC patients with functional magnetic resonance imaging (fMRI) using a passive hierarchical auditory language paradigm (16). The fMRI findings were correlated with the level of the metabolism [obtained with 8F-fluoro-2-deoxyglucose positron emission tomography (FDG-PET)] and the degree of the preservation of the brainstem auditory evoked potentials (BAEPs). Here, we extend our previous results in the same group of DOC patients by assessing the degree of impairment of the left superior temporal gyrus (STG) and of the left arcuate fasciculus, and the level of the metabolism of each hemisphere obtained with FDG-PET. These new findings are considered together with the previously obtained results, with a special reference to the clinical sub-categorization of MCS patients as MCS+ or MCS−.

## 2. Methods

The clinical, neurophysiological, fMRI, and FDG-PET procedures are briefly reported in this section, as they were extensively described in our previous work (16).

Here, we complemented these information with the proposed subcategorization of MCS patients (13) (i.e., MCS+ and MCS−) and with additional data about the integrity of the left STG, investigated with structural magnetic resonance imaging (sMRI), and of the left arcuate fasciculus, investigated with diffusion tensor imaging (DTI) and the FDG-PET values of the left and right hemisphere. The time interval between the neuroimaging/neurophysiological recordings and the closer in time behavioral assessment was <24 h.

### 2.1. Participants

We recruited a consecutive sample of DOC patients hospitalized for a 1-week program of multimodal assessment at the Coma Research Center at the Fondazione IRCCS Istituto Neurologico Carlo Besta of Milan (17, 18). Patients exclusion criteria for this study were as follows: any contraindication to perform neuroimaging examinations, the absence of left and right BAEPs, and extended anatomical lesions in bilateral STG, as detected in sMRI. The initial sample comprised 14 Italian patients with DOC (4 VS and 10 MCS); however, due to excessive head movements during the MRI scanning session, 3 MCS patients were discarded from the subsequent analyses (19). The final sample of patients with DOC included 4 VS, 5 MCS−, and 2 MCS+ (4 males; median age: 57 years, range: 19–69 years; etiology: 4 traumatic brain injury, 5 hemorrhagic brain injury, 2 anoxic brain injury; median time post-injury: 27 months, range: 5–252 months). A summary of the clinical information is reported in Table 1. The local Ethics Committee approved all the aspects of this research and written informed consent was obtained from the legally authorized representative of the patients and from healthy participants (see below) prior to their inclusion in the study. The multimodal evaluation comprised BAEPs, sMRI, DTI, fMRI during the administration of a passive hierarchical auditory language paradigm, and FDG-PET. The clinical assessment of the patients was performed with the CRS-R (2), and MCS patients were further sub-categorized in MCS+ and MCS− according to the proposal of Bruno et al. (13). In addition to the group of healthy participants who underwent the fMRI study and described in the previous paper (16), a group of 20 healthy control (HC) participants (12 females median: 44 years; range: 23–66 years; all right-handed) underwent an MRI DTI study. All the participants of both control groups (fMRI and DTI control groups) declared themselves healthy with no history of neurological or psychiatric diseases or auditory disorders. Exclusion criteria were the presence of any contraindication to perform MRI acquisitions. Conventional MRI acquisition did not reveal brain abnormalities in any of these individuals.

**Table 1:** Summary of the clinical information of disorders of consciousness patients.

**Patient ID**	**Age (ys)**	**Sex**	**Etiology**	**Time post-injury (mo)**	**CRS-R score**	**CRS-R subscores: A-V-M-O-C-Ar**	**Brain lesions**
VS/UWS1	52	M	ABI	17	7/23	1-1-2-1-0-2	Bil. diffuse cortical GM & WM, cerebellum, brainstem, basal ganglia
VS/UWS2	66	F	ABI	14	7/23	2-0-2-1-0-2	Bil. diffuse cortical GM & WM, cerebellum, brainstem, basal ganglia
VS/UWS3	38	M	TBI	252	8/23	2-1-2-1-0-2	Bil. frontal, diffuse WM, CC, brainstem, bil. th
VS/UWS4	57	F	HBI	5	8/23	2-1-2-1-0-2	L basal ganglia and external capsule, CC
MCS1	69	F	HBI	15	10/23	2-3-2-1-0-2	Bil. fronto-parietal, diffuse WM (+right), midbrain, bil. th
MCS2	57	F	HBI	47	9/23	1-3-2-1-0-2	L fronto-temporo-parietal, brainstem, basal ganglia, L th, CC
MCS3	52	M	TBI	179	9/23	1-3-2-1-0-2	Bil. WM (+right), CC, R th
MCS4	64	F	HBI	32	9/23	1-3-2-1-0-2	L fronto-temporal-parieto-occipital, cortical GM & WM, bil. th (+left)
MCS5	19	F	HBI	6	12/23	4-3-2-1-0-2	Bil. frontal and parietal (+left), CC
MCS6	61	M	TBI	103	10/23	2-3-2-1-0-2	Bil. frontal (+left), CC
MCS7	22	F	TBI	27	9/23	3-1-2-1-0-2	Bil. fronto-temporo-parietal, L occipital, CC, brainstem

*ys, years; mo, months; M, male; F, female; ABI, anoxic brain injury; TBI, traumatic brain injury; HBI, hemorragic brain injury; CRS-R, coma recovery scale-revised (2); A, auditory subscale score; V, visual subscale score; M, motor subscale score; O, oromotor subscale score; C, communication subscale score; Ar, arousal subscale score; Bil., bilateral; L, left; R, right; CC, corpus callosum; th, thalamus*.

### 2.2. Neurophysiological and FDG-PET Assessment

Left and right BAEPs were qualitatively evaluated as absent, altered, and normal, as described in Nigri et al. (16). For each patient (except patient VS4 who did not underwent FDG-PET for technical reason), mean standardized uptake values (SUVs) were derived from FDG-PET for the left and right hemisphere as described in Nigri et al. (16).

### 2.3. MRI Assessments

Neuroimaging data were obtained with a 3T MR scanner (Achieva, Philips Healthcare BV, Best, NL) equipped with a 32-channel head coil. The MR protocol comprised the following structural images (sMRI): a high-resolution 3D TFE T1-weighted, sagittal T1-weighted turbo spin echo (TSE) inversion recovery (IR), axial T2-weighted TSE, and coronal fluid attenuated inversion recovery (FLAIR). Moreover, a whole-brain T2*-weighted echo-planar imaging (EPI) sequence for fMRI (40 axial slices, TR = 2,500 ms, TE = 30 ms, FOV = 240 × 240 mm, gap = 0.5 mm, voxel size = 3 mm^3^, flip angle = 90°, dynamic scans = 245, SENSE = 2.5) and a single-shot EPI sequence for diffusion imaging (70 axial slices, 64 diffusion weighted DWI volumes with independent noncollinear directions and 1 un-weighted volume, TR = 3,900 ms, TE = 70 ms, matrix size = 112 × 112; gap = 0.2 mm; voxel = 2 mm^3^, flip angle = 90°, b = 1,000 s/mm^2^, SENSE = 2) were acquired.

#### 2.3.1. sMRI

The ratings of the left STG were performed on the sMRIs. Two expert neuroradiologists (MB and LD) evaluated the severity of the left STG anatomical and signal abnormality, blinded to patients diagnosis, according to Rosazza et al. (12), using the following scale: 0 (severely damaged, i.e., parenchyma obliterated and/or intense, pervasive hyperintensity), 1 (recognizable but distorted morphology and/or severe signal abnormality), 2 (moderate anatomical damage and/or signal abnormality), 3 (mild anatomical damage and/or signal abnormality), and 4 (normal-appearing). Intra-class correlation coefficient (ICC) (R version 3.5.1, Package “irr”) showed that the degree of the inter-rater agreement between these the two experts raters was very high (ρ = 0.95).

#### 2.3.2. fMRI

In the present study, we employed a passive acoustic version of the semantic priming paradigm to determine the extent of the retained lexico-semantic processing (perceiving and recognizing words and understanding their meaning) (20). Briefly, a total of 120 Italian words (60 used as primes and 60 as targets) and 120 legal pseudowords (60 used as prime and 60 used as target) was used to create 240 pairs of stimuli of (1) associatively related words, (2) unrelated words, (3) word–pseudoword, and (4) pseudoword–pseudoword. Prime and target words were matched for written and spoken frequency, concreteness, imageability, length, and the number of syllables (16).

Each trial of the fMRI paradigm consisted of a pair of auditory stimuli: the prime and the target. Based on the type of prime and target, four different trials were identified: associatively related trials (WWr); unrelated-words trials (WWur); word–pseudoword trials (WP) and pseudoword–pseudoword trials (PP). After the data preprocessing and the application of the general linear model, the low-level contrast (WWr+WWur+WP+PP >baseline condition) and the high-level contrast (WP + PP >WWr+WWur) were computed (*p* < 0.001 uncorrected; cluster size >5voxels). The patterns of activation related to low-level contrast and high-level contrast were consistently replicated in each healthy participant (16); therefore, they were used by an expert neuroradiologist (LD), blinded to patients diagnosis, to classify the fMRI activity of each patient based on the following scale: 0, no activation for the low-level contrast; 1, left-lateralized activity only for the low-level contrast or right-lateralized activity for low-level or high-level contrast; 2, left-lateralized activity for both the low-level and high-level contrasts.

#### 2.3.3. DTI

DTI analyses were performed using ExploreDTI (21) for pre-processing and Trackvis (22) for tractography. In every single participant, a pre-processing pipeline was applied to diffusion-weighted images (DWI): realignment of all volumes to the first non-DWI volume, correction for motion and eddy current distortions, and resampling of diffusion images to a voxel size of 1.5 mm^3^. The T1-weighted image was co-registered to non-DWI volume. On realigned DWI images, a non-brain tissue removal step and nonlinear tensor fitting procedure with robust estimation (23) were applied. Data were then analyzed in the native space. After this step, the fractional anisotropy (FA) maps were obtained. To evaluate the degree of integrity of the left arcuate fasciculus, deterministic whole brain tractography was performed on DWI images, using the Fiber Assessment by Continuous Tracking (FACT) method (23) with the following settings: FA tracking value>0.2, maximum, turning angle at 30°, and fiber length range of 50–500 mm (24). The following procedure was then applied to identify the arcuate fasciculus on the left hemisphere. A two-regions of interest (ROIs) approach was used to identify the three segments of the arcuate fasciculus (25, 26): long direct segment between Wernicke's (ROI in superior posterior temporal cortex) and Broca's (ROI in posterior inferior frontal cortex) territories, the anterior indirect segment between Geschwind's (ROI in inferior parietal lobule) to Broca's territories, and the posterior indirect segment between Wernicke's (ROI in superior posterior temporal cortex) to Geschwind's territories (ROI in inferior parietal lobule). Seeds were manually drawn on FA maps by the same expert operator (AN). Tractography algorithm was used to compute the streamlines between the identified couples of ROIs. The obtained segments were manually corrected by means of exclusion ROIs and saved as binary masks. These masks were then merged to obtain the whole arcuate fasciculus. When there was at least the presence of a single segment of the arcuate fasciculus (anterior, posterior, or longitudinal), the left arcuate fasciculus was considered *reconstructed*, otherwise *non-reconstructed*.

### 2.4. Multimodal Evaluations

Statistical index Kendall's tau -b and recursive portioning approach CART (27) (implemented with R using package rpart) were used to identify predictors most correlated with the CRS-R auditory function subscale. The following predictors were evaluated: left and right BAEPs qualitative evaluation (absent, altered, normal), left STG sMRI score (0–4), left arcuate fasciculus DTI evaluation *(non-reconstructed, reconstructed)*, fMRI score (0,1,2), left and right hemisphere mean SUVs. For the description of the multimodal evaluations, we grouped the patients according to the 3 profiles identified with the CRS-R auditory function subscale scores (2), namely the presence of the *auditory startle* (CRS-R = 1), the *localization to sound* (CRS-R = 2), and the *reproducible or consistent movement to command (CRS-R >2)*.

## 3. Results

**Figure 1:**
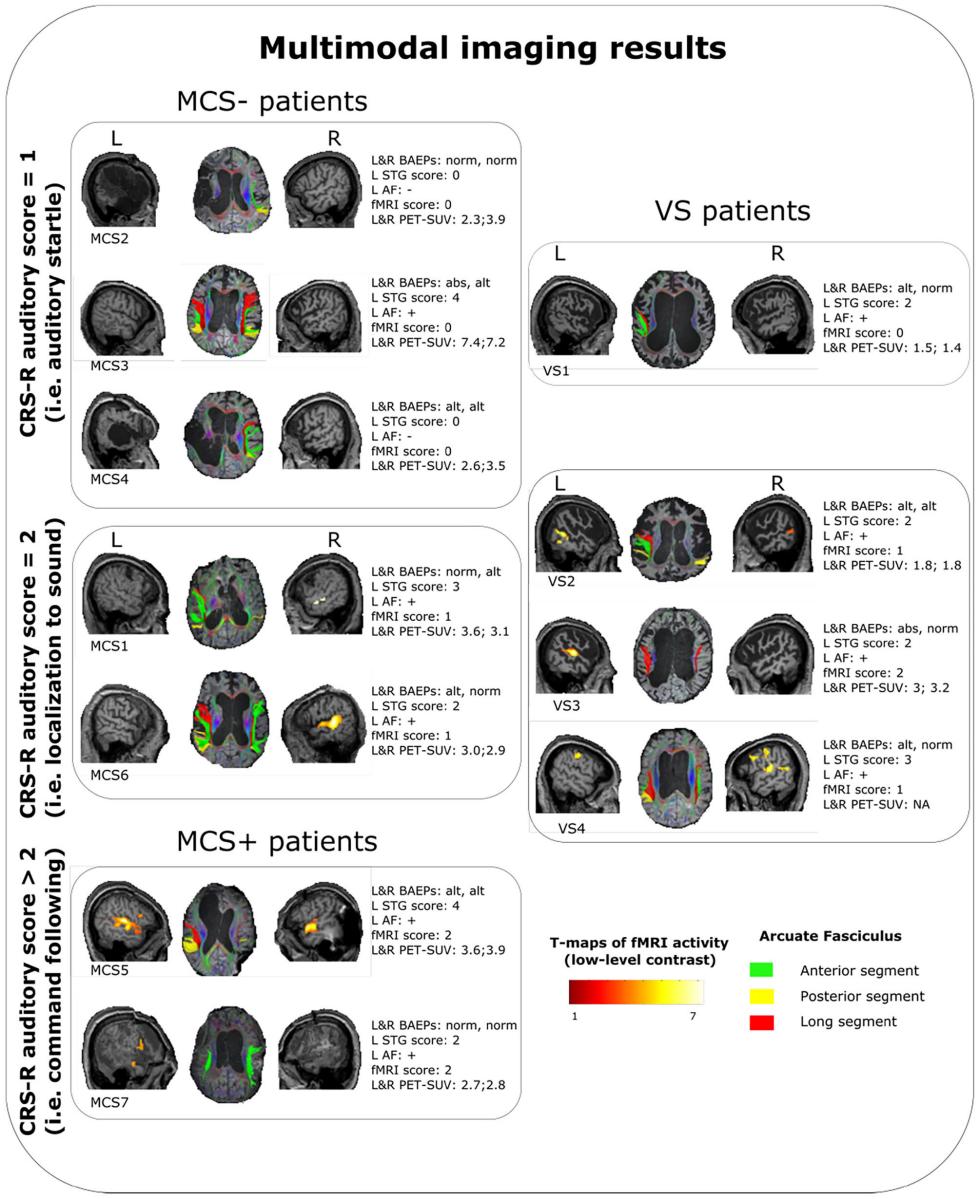
Summary of the multimodal imaging evaluation results for each patients. T-maps of the fMRI results are relative to the low-level contrast (WWr + WWur + WP + PP > baseline condition). Left arcuate fasciculus was considered as reconstructed (+) when at least one segment was identified with the used parameters, otherwise as nonreconstructed (−). VS, vegetative state; MCS, minimally conscious state; CRS-R, coma recovery scale-revised; L, left; R, right; STG, superior temporal gyrus; AF, arcuate fasciculus; fMRI, functional magnetic resonance imaging; PET-SUV, positron emission tomography standardized uptake value.

A summary of the results is reported in Figure 1, Table 2.

**Table 2:** Summary results: patients were grouped according to the CRS-R auditory subscale scores.

**ID**	**Diagnosis**	**BAEPs**	**STG**	**AF**	**fMRI**	**PET-SUV**
	*CRS-R auditory score = 1 (auditory startle)*					
VS1	VS	Alt, Norm	2	+	0	1.54; 1.40
	*CRS-R auditory score = 2 (localization to sound)*					
VS2	VS	Alt, Alt	2	+	1	1.79; 1.81
VS3	VS	Abs, Norm	2	+	2	3.02; 3.23
VS4	VS	Alt, Norm	3	+	1	NA
	*CRS-R auditory score = 1 (auditory startle)*					
MCS2	MCS−	Norm, Norm	0	–	0	2.31; 3.95
MCS3	MCS−	Abs, Alt	4	+	0	7.42; 7.20
MCS4	MCS−	Alt, Alt	0	–	0	2.61; 3.46
	*CRS-R auditory score = 2 (localization to sound)*					
MCS1	MCS−	Norm, Alt	3	+	1	3.57; 3.12
MCS6	MCS−	Alt, Norm	2	+	1	3.01; 2.94
	*CRS-R auditory score >2 (reproducible or consistent movement to command)*					
MCS7	MCS+	Norm, Norm	2	+	2	2.73; 2.80
MCS5	MCS+	Alt, Alt	4	+	2	3.6; 3.94

*BAEP, brainstem auditory evoked potentials; VS, vegetative state; MCS, minimally conscious state; Abs, absent; Alt, altered; Norm, normal; STG, superior temporal gyrus integrity; AF, arcuate fasciculus; fMRI, functional magnetic resonance imaging scores; PET-SUV, positron emission tomography standardized uptake values; hem, hemisphere; l, left; r, right; NA, not applicable*.

### 3.1. Neurophysiological and FDG-PET Results

With regard to the neurophysiological evaluations, 1 patient (MCS−) showed altered BAEPs on the right side and no evidence of BAEPs on the left side, three patients (1 VS, 1 MCS−, 1 MCS+) presented left and right altered BAEPs, and 7 patients (3 VS, 3 MCS−, 1 MCS+) presented normal BAEPs at least on one side. BAEPs evaluations and mean SUVs of the left and right hemisphere are reported for every patient in Table 2.

### 3.2. MRI Results

Except in 2 MCS− patients, (left STG sMRI score = 0), in the remaining sample (2 MCS+, 3 MCS− and 4 VS) the left STG sMRI score was >1 (Figure 1). During the administration of the fMRI task, 1 VS and 3 MCS− patients presented no detectable activation for the low-level contrast (fMRI score = 0), 2 VS presented a left-lateralized activity for the low-level contrast (fMRI score = 1), 2 MCS− presented a right-lateralized activity (fMRI score = 1), 2 MCS+ and 1 VS presented a left-lateralized activity for the low-level contrast and for the high-level contrast (fMRI score = 2). In 2 MCS− patients, the left arcuate fasciculus was considered as *non-reconstructed*, while in the remaining sample (2 MCS+, 3 MCS−, and 4 VS) it was considered as *reconstructed* (i.e., reconstruction of at least one segment of the fasciculus). All the HC subjects presented the anterior and posterior indirect segments of the left arcuate fasciculus; the left long direct segment was found in 19 out of 20 (95%) individuals.

### 3.3. Multimodal Evaluations Results

Kendall's tau-b coefficient showed a very strong correlation between the fMRI score and the CRS-R auditory subscale (=0.92). Consistently to this strong correlation, fMRI score was selected by the CART approach as the best predictor satisfying the splitting criterion in the first and second knots. The remaining variables did not show evidence of correlation with the CRS-R auditory function subscale.

#### 3.3.1. Patients Presenting “Auditory Startle” Only (CRS-R Auditory Score = 1)

One VS and 3 MCS (in MCS− condition) patients showed the *auditory startle* at the CRS-R auditory function subscale. Remarkably, these patients did not show any evidence of fMRI-related activity. In the VS patient (VS1), the lack of fMRI-related activity was possibly supported by a very low-level metabolism (as indicated by the lowest SUV observed across the whole sample), since there were a relative preservation of the brainstem auditory pathways and of the core structures of language processing (left STG and arcuate fasciculus). In MCS− patients, the lack of fMRI-related activity was possibly sustained by severe anatomical damage of the left-sided investigated structures (in MCS2 and MCS4 patients) or by a very severe impairment of the brainstem auditory pathways (in MCS3 patient). Notably, this latter patient had a global brain metabolism well preserved (left hemisphere SUV = 7.42, right hemisphere SUV = 7.22).

#### 3.3.2. Patients Presenting “Localization to Sound” (CRS-R Auditory Score = 2)

Three VS and 2 MCS (in MCS− condition) patients presented the *localization to sound* at the CRS-R auditory function subscale. Remarkably, these patients presented a relative integrity of BAEPs (qualitative assessment: from altered to normal) and of the core structures of language processing (left STG scores: from 2 to 3; left arcuate fasciculus: reconstructed) always associated with a certain degree of fMRI-related activity. In particular, the VS patients of this category presented low-level (VS2, VS4) or high-level left-lateralized activity (VS3), while MCS patients presented a low-level left-lateralized (MCS1) activity or right-lateralized activity (MCS6). Also in this category, the cerebral metabolism was quite variable (SUVs range: 1.8–3.6), with a VS patient (VS3) showing a metabolism in the left hemisphere (SUVs = 3.02) superior to some MCS patients (MCS2, MCS4, MCS7).

#### 3.3.3. Patients Presenting “Reproducible or Consistent Movements to Command” (CRS-R Auditory Score >2)

The patients of this subgroup (2 MCS+) presented or altered bilateral BAEPs accompanied by an integrity of the core structures of language processing (patient MCS5) or a complete preservation of bilateral BAEPs accompanied by a relatively low integrity of the left STG and by the presence of the left arcuate fasciculus (patient MCS7). Consistent with the behavioral profile, both patients presented high-level left-lateralized fMRI activity.

## 4. Discussion

In the present study, we investigated the degree of the integrity of the main cerebral structures (left STG and left arcuate fasciculus) and functions (BAEPs, mean SUVs of the left and right hemisphere, fMRI activity during the execution of a hierarchical language task) involved in auditory language processing in a sample of patients with DOC. Our results support the hypothesis of a relationship between the level of the preservation of the investigated structures and functions and the CRS-R auditory subscale scores (2). In the following, we will discuss our findings in the framework of the patients behavior as observed at the CRS-R auditory subscale.

### 4.1. Patients Presenting “Auditory Startle” Only

In our sample, the patients presenting only the *auditory startle* (3 in MCS− and 1 in VS) at the behavioral level (CRS-R auditory subscale score = 1), showed no evidence of fMRI activity (fMRI score = 0) during the administration of the auditory linguistic paradigm. Importantly, in a one-to-one correspondence, the patients exhibiting no fMRI activity presented only the *auditory startle* at the CRS-R auditory subscale. These observations support the hypothesis that these patients were unable to differentiate, at the neural level, the noise of the scanner from the auditory linguistic stimuli and vice versa. Importantly, in all the 3 MCS patients of this sub-group, the presence of the *auditory startle* and the associated lack of fMRI activity were linked to an important alteration of the bilateral BAEPs (absence and altered BAEPs; absence of both BAEPs was an exclusion criteria for our study), or to severe alterations of core structures of language processing in the receptive domain (left STG and left arcuate fasciculus). This suggests a correspondence between the behavior of the *auditory startle* and the presence of structural and functional impairment in the processing of auditory linguistic stimuli. Notably, the observed behavioral phenotypes seemed to be independent from the level of the global cerebral metabolism, as indicated by the presence, in this subgroup, of patients with high SUVs (e.g., MCS3 patient). The only VS patient (VS1) belonging to this subgroup presented a relative integrity of BAEPs and of the core structures of language processing (left STG and left arcuate fasciculus), but showed the lowest SUV across the whole investigated sample. It is very plausible, therefore, that the very low-level metabolism was at the basis of the observed behavioral and fMRI profile of this patient.

### 4.2. Patients Presenting “Localization to Sound”

The patients (2 MCS− and 3 VS) presenting the ability to localize sounds at the CRS-R auditory subscale (score = 2) showed evidence of fMRI activity during the administration of the language paradigm. Remarkably, these patients, except one (patient VS3), presented a fMRI score = 1 (left-lateralized activity in the low-level contrast or right-lateralized activity in the low-level or high-level contrasts). They also presented, in agreement with the fMRI profile, a relative preservation of the BAEPs and of the core structures of language processing. The observed fMRI activity suggests that these patients were able, at neural level, to differentiate the linguistic stimuli from the noise of the scanner, but they were unable to differentiate between words and pseudo-words. The behavior of *localization to sound* at the CRS-R auditory subscale seems thus to be consistent with these neuroanatomical and neurofunctional findings. The presence of the fMRI activity, although of low-level, and the relative preservation of BAEPs and of the investigated structures (i.e., left STG and left arcuate fasciculus) suggest that, in this subgroup, the observed behavior was not related to a specific impairment in language processing but to the level of global brain metabolism. The employed semi-quantitative approach (i.e., SUV), however, does not allow to make strong claims about this hypothesis. Notably, in this sample, we observed 1 VS patient (VS3) showing high-level fMRI activity (fMRI score = 2), indicating, very plausibly, a preservation of automatic lexical processing. Interestingly, this patient showed also the highest SUV among the investigated VS patients. It is important to note that 1 MCS patient (patient MCS6), reported as right-handed, showed a different pattern of fMRI activity when processing words and pseudowords lateralized to the right hemisphere. However, based on the evidence that the 93% of right-handed individuals presents left-hemisphere language dominance (28), we consider this patient as unable to differentiate between words and pseudowords.

### 4.3. Patients Presenting “Reproducible or Consistent Movement to Command”

The behavior of *reproducible or consistent movement to command* on the CRS-R auditory function subscale (score >2) was observed in 2 MCS patients (classified as MCS+). As expected, they showed a relative preservation of BAEPs and of the investigated neuroanatomical structures, associated with a high-level fMRI activity lateralized to the left hemisphere. Consistently, no MCS− patients showed high-level fMRI activity.

The subcategorization proposed by Bruno et al. (13, 14) classifies MCS patients as MCS+ when they present high-level behavioral responses, such as command following, intelligible verbalizations or nonfunctional communication, while as MCS− when they present low-level behavioral responses, such as visual fixation and pursuit, automatic motor reactions, and localization to noxious stimulation. This important clinical subclassification is still under scrutiny, as it is unclear if it mainly reflects different levels of residual language abilities and/or different levels of consciousness. Remarkably, previous studies have shown that this subcategorization is related to different functional and anatomical neural substrates related to language processing: MCS−, in comparison to MCS+, were shown to be characterized by impaired metabolism and severe anatomical abnormalities in the left cortical networks, comprising Broca's and Wernicke's areas (14). More recently, Aubinet et al. (15), employing resting-state fMRI, showed that MCS− patients present lower functional connectivity in the language control network (i.e., left fronto-parietal network). Our study, directly probing the language system with a multimodal approach, suggests that the distinct behaviors exhibited by MCS patients at the CRS-R auditory subscale might rely on distinct neuro-anatomical and functional substrates.

MCS+ patients showed neural activity differentiating words from pseudowords, as indicated by the different patterns of left-lateralized fMRI activity during the administration of these two different categories of stimuli. Consistently, this high-level fMRI activity was supported by the relative preservation of the brainstem auditory function and of the core structures involved in language processing (left STG and fasciculus arcuate) and, possibly, by a relatively high-level cerebral global metabolism. Indeed, although the relationship between fMRI activity and global cerebral metabolism is still far from being completely solved, it is clear that a strong link between the two exists (29). All these neural features are in agreement with the high-level behavioral profile, typical of MCS+ patients, and support the hypothesis of an efficient auditory/language processing in these patients.

MCS− patients exhibiting the *auditory startle* only response (CRS-R auditory subscale score = 1) presented severe alterations of BAEPs or of the core structures of language processing and, as a possible consequence of these severe abnormalities, no evidence of fMRI activity. A possible, although important, clinical consequence of these findings is that MCS− patients showing the *auditory startle* might be prevented from the comprehension of spoken language because of the impairment of auditory language processing, independently from the level of the metabolism. Remarkably, we do not know whether this behavior (i.e., auditory startle) might indicate also MCS− patients with an integrity of the structures and functions of language processing, but with a relatively low-level global cerebral metabolism. Although we did not find such a neural profile, the small size of the investigated sample does not allow to exclude this possibility. However, in our opinion this may be unlikely: it is plausible that in MCS condition the relatively sustained global metabolism may allow at least the localization to sound during the administration of the CRS-R in patients with a relative integrity of BAEPs and of the core structures of language processing, as observed in our study.

On the other hand, MCS− patients exhibiting *localization to sound* (CRS-R auditory scale score = 2) presented a relative preservation of BAEPs and of the core structures of language processing, with no ability to differentiate, at neural level, words from pseudowords, as shown by the low-level fMRI activity. The observed neural profiles suggest that MCS− patients showing the *localization to sound* might be characterized by a relative preservation of the language processing, despite the low-level fMRI activity. It is very plausible that the inability to differentiate at neural level words from pseudowords might rely on low-level global cerebral metabolism, which has been shown, in its absolute values, to be a marker of the level of consciousness (30). Based on this line of reasoning, we speculate that these patients do not present an efficient language processing because of a lower level of consciousness in comparison to MCS+ patients.

Also in regard to VS, it is possible to make some speculation. VS patients may present at the CRS-R auditory subscale, for definition, the *auditory startle* or the *localization to sound*. In our sample, the only VS patient presenting the *auditory startle* showed a relative integrity of BAEPs and of the core structures of language processing, with no evidence of fMRI activity, associated with the lowest SUV computed in our sample. In this case, it is plausible that the behavioral profile was related to the low-level of consciousness. In principle, it should be possible to observe also patients, diagnosed as VS, presenting severe alterations of BAEPs and/or of the core structures of language processing, exhibiting a similar behavioral profile (i.e., auditory startle). If this is the case, it is clear that these patients might be vulnerable to misdiagnosis, being possible that the linguistic disturbance might mask possible conscious behavior.

### 4.4. Limits of the Study

The main limit of the study is the relatively small and heterogeneous (for different etiology and time post-injury) sample of patients with DOC. However, the extended neuroimaging assessment allowed to map in unprecedented way in these patients the residual language processing. Another limit is the absence of patients with bilateral STG lesions and negative BAEPs who could have served as patient controls to detect false positive results. The confirmatory evidence of aphasia (i.e., external validation) is an intrinsic limit related to this disorder. Another limit of the study is the use of the semiquantitative method (i.e., SUV) to compute the level of global metabolism. This method does not allow to make strong claims about the level of consciousness of the investigated patients, as in the case of quantitative methods (30, 31).

The investigation of the function and morphology of the STG alone among the diverse language-related areas (e.g., left inferior frontal gyrus, middle temporal gyrus) (32) might be considered another limit of the study. Indeed, during the recovery from aphasia, fully aware aphasic patients present, in the acute phase, reduced activation of the diverse left-hemisphere language areas, in the subacute phase, maladaptive recruitment of the homologous right-hemisphere regions, and then, in the chronic phase, the normalization of the activity of the left-hemisphere language areas (33). Therefore, it is possible to speculate that DOC patients, when impaired in their language function, might spontaneously reorganize similarly, considering, however, the limits imposed by the cortical low-level metabolism (30, 31), and by the localization and extension of the insult. Despite these observations, we chose to limit our investigation to STG: in our previous work, we have shown that a sample of healthy participants (*n* = 18) presented, for both low-level and high-level contrast, robust activity in STG during the employed fMRI task, which capitalized on the repetition priming effect (20). Although it is not possible to exclude plasticity phenomena that could allow other brain regions to support linguistic processing in long-term DOC patients, our results support the thesis that the level of retained lexical processing can be inferred by the activity of the STG during this specific fMRI task.

## 5. Conclusion

Our findings indicate that, in MCS patients, distinct behaviors at the CRS-R auditory function subscale might be sustained by different neurofunctional profiles. In particular, our results support the hypothesis that MCS− patients presenting the *auditory startle* might be aphasic in the receptive domain, independently from the level of cerebral metabolism and, therefore, from the level of consciousness (30). Moreover, our results support the hypothesis that MCS− patients exhibiting the *localization to sound* might present a lower level of consciousness in comparison to MCS+ patients, because, although presenting a relative integrity of BAEPs and of the core structures of language processing, they show low-level fMRI activity, in which neurophysiological bases are in the level of the metabolism, that seems to sustain the level of consciousness (30).

Importantly, based on our results, we speculate that patients diagnosed as VS and presenting the *auditory startle* are a particularly vulnerable population at risk of misdiagnosis. On the one side, they can present a low level of metabolism and possibly of consciousness, as the patient observed in our study, but, on the other side, in the case of important BAEPs alterations and/or severe abnormalities of the left STG or left arcuate fasciculus, they might suffer from aphasia in the receptive domain, as MCS− patients presenting the auditory startle in our investigated sample.

It is important to note that, in this study, we investigated a sample of DOC patients with time post-injury >5 months, therefore our results might not be valid in patients in acute DOC.

Our findings add further evidence to the hypothesis that language disorders in the receptive domain are crucial in the clinical assessment of DOC patients. If replicated in a wider sample of DOC patients, our results might have important repercussions in the clinical setting.

## Data Availability Statement

The datasets generated for this study are available on request to the corresponding author.

## Ethics Statement

The studies involving human participants were reviewed and approved by IRB of Fondazione IRCCS Neurologico Carlo Besta, Milan. The legally authorized representative of the patients and healthy participants provided their written informed consent.

## Author Contributions

SF, SC, and MB conceived the study. DRS, DD, and EV oversaw collection and interpretation of BAEPs data. DS and ML collected the clinical data. LD'I, MB, SF, GD, and AN oversaw collection and clinical interpretation of MRI data. GM oversaw collection and analyses of PET data. SF, AN, CR, EC, and GD carried out the fMRI and DTI analysis presented in the paper. SF, SC, MB, and ML interpreted the results and drafted the manuscript. ML and MB secured funding. All authors provided critical feedback on the manuscript.

## Conflict of Interest

The authors declare that the research was conducted in the absence of any commercial or financial relationships that could be construed as a potential conflict of interest.
