# Corrigendum: Preservation of Language Processing and Auditory Performance in Patients With Disorders of Consciousness: A Multimodal Assessment

**DOI:** 10.3389/fneur.2021.656747

**Published:** 2021-02-16

**Authors:** Stefania Ferraro, Anna Nigri, Ludovico D'Incerti, Cristina Rosazza, Davide Sattin, Davide Rossi Sebastiano, Elisa Visani, Dunja Duran, Giorgio Marotta, Greta Demichelis, Eleonora Catricala', Sonja Kotz, Laura Verga, Matilde Leonardi, Stefano Cappa, Maria Grazia Bruzzone

**Affiliations:** ^1^School of Life Science and Technology, MOE Key Laboratory for Neuroinformation, University of Electronic Science and Technology of China, Chengdu, China; ^2^Neuroradiology Department, Fondazione IRCCS Neurologico Carlo Besta, Milan, Italy; ^3^Neurology, Public Health, Disability Unit and Coma Research Centre, Fondazione IRCCS Neurologico Carlo Besta, Milan, Italy; ^4^Department of Neurophysiology and Diagnostic Epileptology Unit, Fondazione IRCCS Neurologico Carlo Besta, Milan, Italy; ^5^Department of Nuclear Medicine, Fondazione IRCCS Ca' Granda Ospedale Maggiore Policlinico, Milan, Italy; ^6^Department of Psychology, Scuola Universitaria Superiore, Pavia, Italy; ^7^Department of Psychology, Maastricht University, Maastricht, Netherlands; ^8^IRCCS Mondino Foundation, Pavia, Italy

**Keywords:** disorders of consciousness, magnetic resonance imaging, brainstem auditory evoked potentials, positron emission tomography, language processing

In the published article, there was an error regarding the affiliations for Stefano Cappa. As well as having affiliation 6, they should also have affiliation 8 - IRCCS Mondino Foundation, Pavia, Italy.

The authors apologize for this error and state that this does not change the scientific conclusions of the article in any way. The original article has been updated.

